# Environmental Context of Hominin Foot Evolution

**DOI:** 10.1002/ajpa.70340

**Published:** 2026-10-02

**Authors:** Yeganeh Sekhavati, Thomas Cody Prang, David Strait

**Affiliations:** ^1^ Department of Organismal Biology and Anatomy University of Chicago Chicago Illinois USA; ^2^ Department of Anthropology Washington University in St. Louis St. Louis Missouri USA; ^3^ Palaeo‐Research Institute University of Johannesburg Auckland Park Gauteng South Africa

**Keywords:** bipedalism, foot, hominins paleoenvironment, synapomorphies

## Abstract

**Objectives:**

This study investigates the environmental contexts that influenced the evolution of hominin foot morphology, focusing on when, where, and under what conditions key synapomorphies evolved. We test whether shifts in ecological conditions are associated with changes in locomotor function.

**Methods:**

We used phylogenetic and biogeographical analyses with paleoenvironmental data to evaluate the relationships between foot morphology and environmental context. Phylogenetic trees were inferred using the fossilized birth‐death model and time‐calibrated. Multiple topologies were used to explore alternative evolutionary hypotheses. Historical biogeography was reconstructed using BioGeoBEARS. Finally, environmental conditions at key ancestral nodes were inferred by linking reconstructed geographical regions with paleoenvironmental proxies.

**Results:**

The results suggest that the origin of bipedalism likely occurred during intervals of increasing aridity. Later in hominin evolution, wetter environments became more important, and most derived traits evolved during wet or mixed environments. The last common ancestors (LCA) of most *Australopithecus* and *Paranthropus* species likely lived in wetter environments, and the LCA of Homo lived in both dry and wet environments. Across all trees, an average of 5.3 synapomorphies evolved in wet environments, 5.0 in dry and wet environments, and 4.3 in mixed environments. The results suggest that a considerable number of foot synapomorphies evolved during periods of ecological transition.

**Conclusions:**

Ecological flexibility seems to be significant in hominin foot evolution, enabling synapomorphies to persist as hominins faced different environmental conditions. These findings illustrate how environments influenced the evolution of key adaptations in hominins.

## 1 Introduction

Reconstructing the habitats of early hominins helps explain how ecological pressures may have influenced locomotor adaptations. Environmental context is important in understanding the origins of bipedalism (Richmond et al. 2001; Pickford 2006; Vrba 2007; Cerling et al. 2011; Senut et al. 2018). Moreover, incorporating environmental variables is essential for scenario building in paleoanthropology and for developing robust hypotheses about the selective pressures underlying the evolution of bipedalism (Tattersall and Eldredge 1977).

The role of paleoclimatic changes on human evolution has been widely debated (Keith 1923; Morton 1935; Washburn 1967; Tuttle 1981; Fleagle et al. 1981; Richmond et al. 2001; Thorpe et al. 2014), with competing hypotheses linking bipedalism to different environmental pressures. We evaluate three hypotheses regarding the environmental context of the early evolution of bipedalism. The Savannah Hypothesis (H1) predicts that bipedal synapomorphies should appear primarily in open, dry habitats close to the root of the hominin clade. The forest hypothesis (H2) predicts that these traits evolved mainly in closed, wet (wooded) habitats and are not linked to aridification. The variability selection hypothesis (H3) proposes that bipedal traits emerge in transitional (changing) or mixed environments and are more common during environmental shifts (e.g., from wetter to drier). To test these hypotheses, we combine phylogenetic ancestral trait evolution, ancestral range reconstructions, and regional paleoenvironmental data. Specifically, we will assess whether environmental conditions and changes align with the timing and phylogenetic placement of key locomotor synapomorphies in early hominins. Each hypothesis predicts different ecological settings for the emergence of bipedal traits: open, dry; closed, wet; or environmentally variable or transitional habitats.

### 1.1 Environmental Context and the Evolution of Bipedalism

Numerous hypotheses have linked the origin of bipedalism to environmental selective pressures. The Savannah Hypothesis explains the origin of bipedalism as a response to late Miocene environmental change, in which forest fragmentation and the spread of open, arid habitats reshaped selective pressures on early hominins (Dart 1925; Vrba 1985; Stanley 1992). Based on this hypothesis, the decline of closed, densely wooded environments coincides with the emergence of bipedal locomotion (Bonnefille 1983; Dupont and Leroy 1995; Cerling et al. 2011). The hypothesis predicts a close temporal match between early bipedal traits and major climatic shifts toward drier, more open landscapes (Bonnefille 1983; Dupont and Leroy 1995; Cerling et al. 2011). The Savanna hypothesis was strongly advocated by Dart (1949) after the discovery of the Taung child (Dart 1925), as it was consistent with his “hunting hypothesis” in which bipedalism evolved for long‐distance viewing over tall grasses (Dart 1949).

The Savanna Hypothesis has been challenged, especially regarding the timing and ecological context of early bipedalism (Potts 1998; WoldeGabriel et al. 2001; deMenocal 2004; White et al. 2009; Bender et al. 2012; Bamford et al. 2013; Blumenthal et al. 2017). The widespread expansion of open, arid environments may have occurred millions of years after the first appearance of bipedal traits, weakening the direct link between savannah formation and the origin of bipedalism (Vrba 2007; Pawłowski and Nowaczewska 2015). This motivates an alternative hypothesis called the Forest Hypothesis, which proposes that bipedalism evolved in closed, wetter, forested habitats and emphasizes arboreal settings rather than open landscapes (Rayner et al. 1993).

The variability selection hypothesis emphasizes climatic instability (Potts 1998; Hardt et al. 2007; Pawłowski and Nowaczewska 2015). In this view, bipedalism did not evolve as an adaptation to open savannahs or closed forests, but as a locomotor strategy that increased resilience across fluctuating environments (Potts 1996, 1998). The hypothesis predicts that bipedal traits should emerge during periods of pronounced climatic variability rather than under prolonged, stable environmental conditions.

Testing these competing hypotheses requires evaluating whether environmental change coincides with the evolution of locomotor synapomorphies (shared derived traits). Therefore, reconstructing the timing and phylogenetic distribution of bipedal traits with paleoenvironmental records allows us to determine whether their evolution aligns with predictions from savannah, forest, and climatic variability hypotheses.

### 1.2 Pattern of Foot Evolution

The hominin foot is central to understanding the evolution of bipedalism. As the point of contact between the body and ground, its morphology provides insight into posture and locomotor efficiency (Harcourt‐Smith and Aiello 2004). Derived features such as midfoot stability, an adducted hallux, and a longitudinal arch are key evolutionary steps toward modern human bipedalism (Latimer et al. 1987; Lovejoy, Latimer, et al. 2009; Prang 2016b).

A recent phylogenetic study has mapped synapomorphies and plesiomorphies across key ancestral nodes in the hominin lineage (Sekhavati et al. 2025). Their analysis compared three alternative cladograms and identified the synapomorphies that evolved at various nodes (Figures 1, 2, 3). Some of those nodes were found in every tree, while others were specific to particular trees. The methods used to derive the trees are described in the next section. Here, we briefly summarize our prior findings (Sekhavati et al. 2025) on the evolution of pedal characters.

**FIGURE 1:**
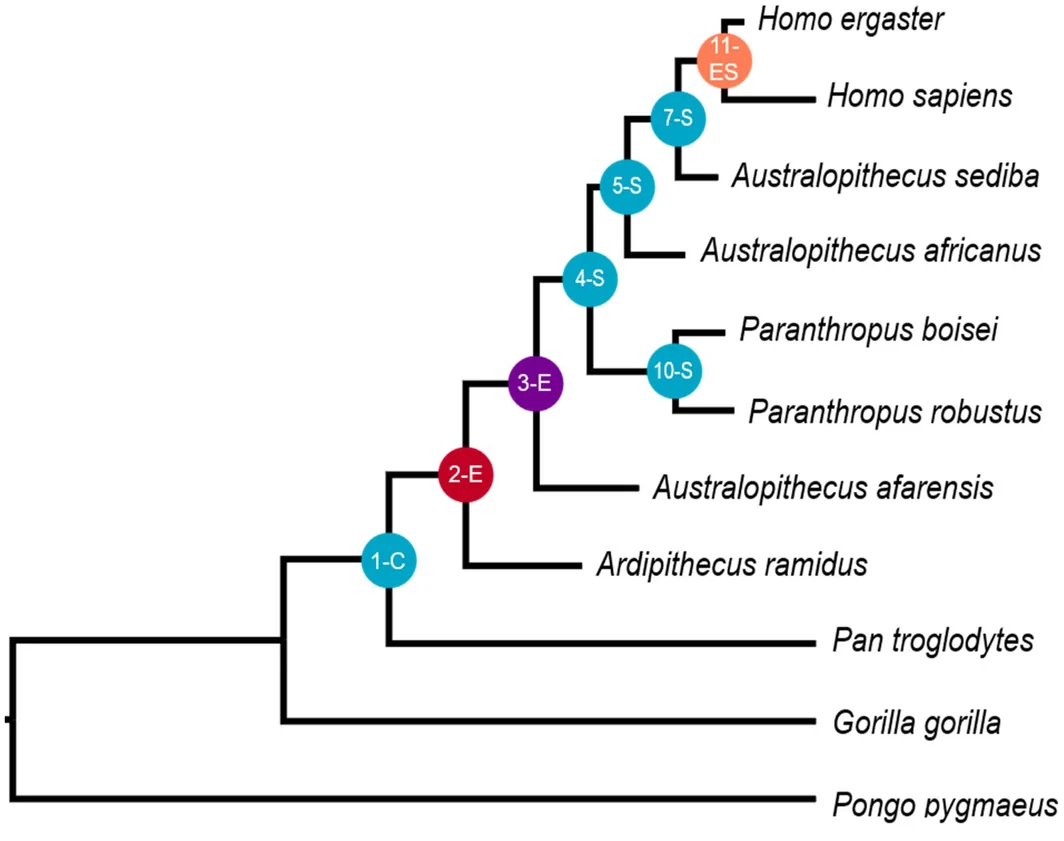
Results for Tree 1, with colors indicating the environment: Blue = wet, red = dry, purple = mixed, and orange = wet and dry. The number at each node represents the node number, followed by the reconstructed biogeography: C = Central Africa, E = Eastern Africa, and S = Southern Africa.

**FIGURE 2:**
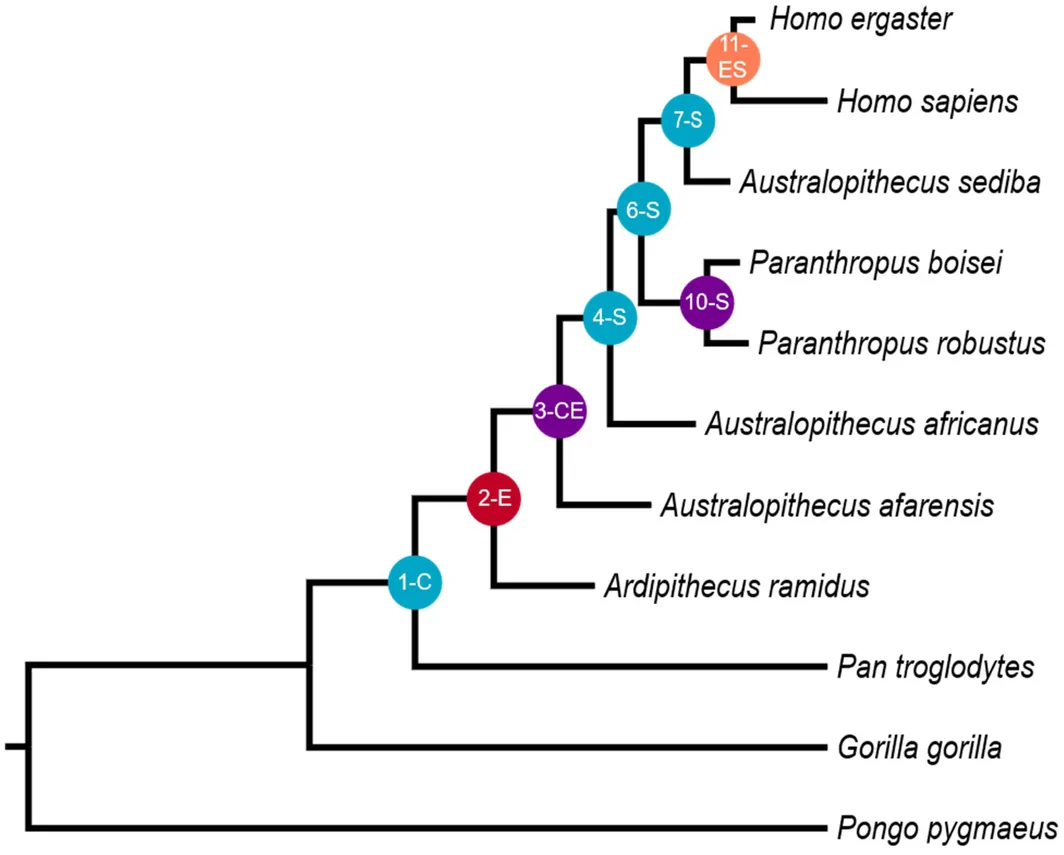
Results for Tree 2, with colors indicating the environment: Blue = wet, red = dry, purple = mixed, and orange = wet and dry. The number at each node represents the node number, followed by the reconstructed biogeography: C = Central Africa, E = Eastern Africa, and S = Southern Africa.

**FIGURE 3:**
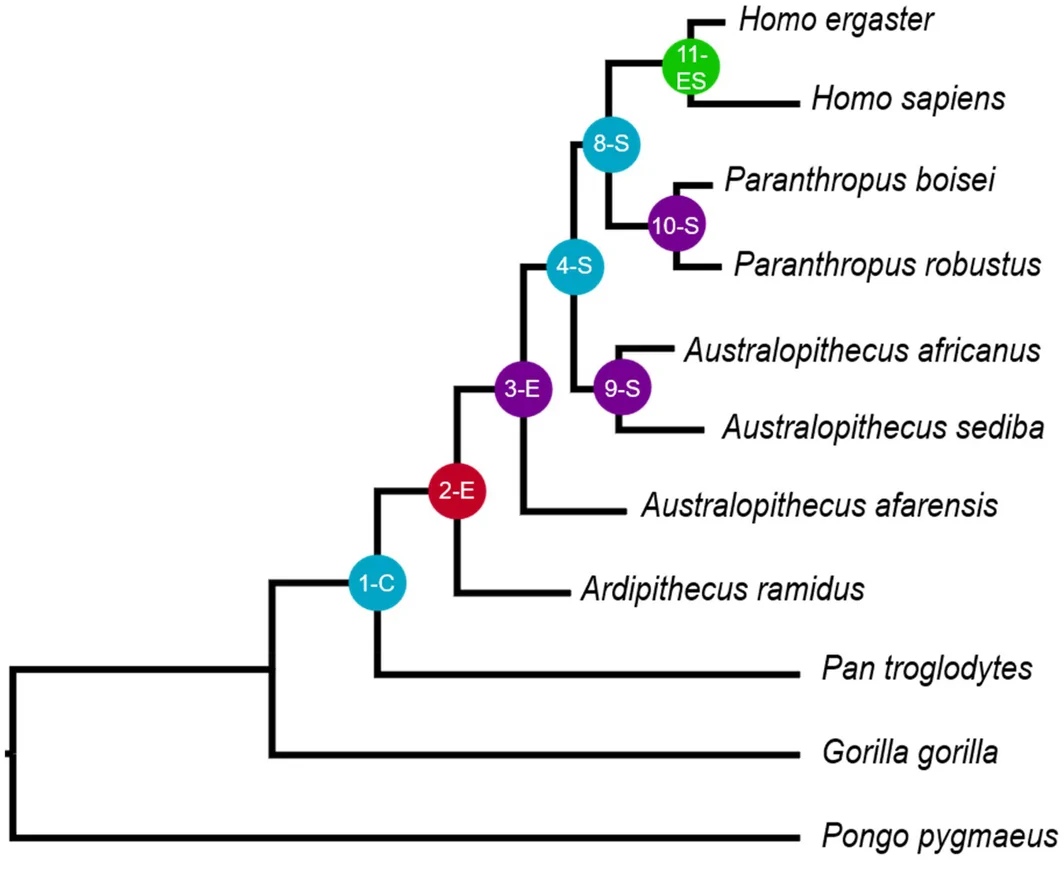
Results for Tree 3, with colors indicating the environment: Blue = wet, red = dry, purple = mixed and green = mixed and wet. The number at each node represents the node number, followed by the reconstructed biogeography: C = Central Africa, E = Eastern Africa, and S = Southern Africa.

The last common ancestor (LCA) of humans and chimpanzees (node 1) likely combined terrestrial heel‐strike plantigrady and vertical climbing (Latimer and Lovejoy 1990a; Gebo 1992, 1996; McNutt et al. 2018; DeSilva et al. 2019; Prang 2019, 2022, 2024; Prang et al. 2025; Sekhavati et al. 2025). Like node 1, the hominin LCA (node 2; Figures 1, 2, 3) retained broadly chimpanzee‐like foot morphology (Latimer et al. 1987; DeSilva 2009; McNutt et al. 2018; Manter 1941; Elftman 1960; Prang 2016a).

The LCA of *Australopithecus* and later hominins (node 3; Figures 1, 2, 3) and the LCA of *Australopithecus africanus*, *Australopithecus sediba*, *Paranthropus*, and *Homo* (node 4; Figures 1, 2, 3) are inferred to have multiple synapomorphies that improve bipedal locomotion (Table 2; Manter 1941; Elftman 1960; Latimer et al. 1987; Latimer and Lovejoy 1990a; Richmond 2007; Congdon 2012; Nguyen et al. 2014; Prang 2016a). Compared with earlier nodes (nodes 1 and 2), these ancestors show traits associated with bipedalism (Lewis 1980; Latimer and Lovejoy 1990a; Aiello and Dean 1990; DeSilva 2008, 2009; Prang 2016a; Sekhavati et al. 2025) while retaining arboreal features (Day and Wood 1968; Latimer et al. 1987; Gebo 1992; Richmond 2007; DeSilva 2009; Rolian et al. 2009; Congdon 2012; Nguyen et al. 2014; Boyle and Desilva 2015; Sekhavati et al. 2025). Across nodes 5, 6, and 8 (Figures 1, 2, 3), additional synapomorphies evolved, further enhancing bipedal function (Weidenreich 1923; Morton 1935; Latimer and Lovejoy 1990a, 1990b; Aiello and Dean 1990; Richmond 2007; Nguyen et al. 2014; Prang 2016b). Table 2 summarizes the synapomorphies associated with each node.

Not all inferred synapomorphies are associated with improved bipedal performance. Synapomorphies associated with foot function in arboreal contexts characterize the LCAs of the *A. africanus* + *A. sediba* clade (present in one of the three trees: node 9; Figure 3) and the *Paranthropus* clade (present in all trees: node 10; Figures 1, 2, 3). These traits may reflect adaptation to forested environments. Arboreal synapomorphies at these nodes suggest that hominin foot evolution did not follow a linear progression toward bipedalism (Sekhavati et al. 2025). Therefore, we hypothesize that arboreal traits were either retained or evolved in association with ecological pressures, such as forested environments or the need for locomotor versatility.

Finally, the LCA of *Homo* (node 11, Figures 1, 2, 3) has the greatest number of synapomorphies (Susman and Brain 1988; Prang 2016b). A longitudinal arch also appears at this node, which is a key adaptation for economical bipedalism (Boyle and Desilva 2015; Pontzer 2017).

Although our previous work (Sekhavati et al. 2025) documented synapomorphies and symplesiomorphies at each node, our present analysis focuses exclusively on synapomorphies. Synapomorphies are evolutionary innovations that define clades and help reconstruct changes in locomotor morphology (Lauder 1990; Lockwood 2007). Connecting them to ecological context could help us in understanding their evolution. Because the foot reflects adaptations to substrate and locomotion, it may offer a sensitive signal of how early hominins responded to shifting habitats (Harcourt‐Smith and Aiello 2004; Rolian et al. 2009).

## 2 Materials and Methods

To examine the effect of the environment on foot evolution, we estimated the geographic locations represented at various internal nodes of the evolutionary tree. We also reconstructed the environmental conditions of those geographic locations at the specific times. To do so, we inferred a time‐calibrated phylogeny, followed by reconstructing hominin historical biogeography (the pattern of how organisms are distributed across the landscape, and when those patterns emerged). Finally, we examined the environmental context during those periods to gain insights into how ecological factors may have influenced foot evolution.

### 2.1 Taxa

We included four extant hominid taxa: *Pan* spp., *Gorilla* spp., *Pongo* spp., *and Homo sapiens*. The fossil sample comprises early hominins found in Africa for which we have foot data and craniodental characters to infer their phylogeny. The sample includes *Ardipithecus ramidus, Australopithecus afarensis, A. africanus, A. sediba, Paranthropus robustus, Paranthropus boisei*, and *Homo erectus* (Table 1). We recognize *A. sediba* as a distinct taxon in our analysis, although alternative taxonomic treatments have been proposed (Martin et al. 2024; see Supporting Information S1).

**TABLE 1:** Taxa included in this study, their age, and geographic ranges.

Taxon	Region(s)	Age (FAD–LAD, in Ma)	Fossil specimens
*Ardipithecus ramidus*	B	4.8–4.3 (Semaw et al. 2005; Simpson et al. 2019)	ARA‐VP‐6/1000, ARA‐VP‐6/505, ARA‐VP‐6/500‐067, ARA‐VP‐6/500‐128, ARA‐VP‐6/500‐023, GWM67/P2
*Australopithecus afarensis*	A, B	3.9–3.0 (Asfaw 1987; Kimbel et al. 1994; Kimbel and Delezene 2009; Deino 2011)	A.L. 333‐147, A.L. 333‐28, A.L. 333‐54, A.L. 333‐115, A.L. 333‐8, A.L. 333‐55, A.L. 333‐79, A.L. 333‐37, A.L. 333‐36, A.L. 333‐133, A.L. 333‐75, A.L. 333‐158, A.L. 333‐47, A.L. 288‐1, A.L. 333‐160, A.L. 333‐13
*Australopithecus africanus*	C	3.0–2.1 (Mcfadden et al. 1979; Partridge et al. 2000; Herries et al. 2013; Pickering and Herries 2020)	StW 88, StW 102, StW 363, StW 347, StW 352, StW 562, StW 595, StW 89, StW 377, StW 238, StW 387, StW 388, StW 435, StW 477, StW 496, StW 114/115, StW 470
*Australopithecus sediba*	C	2.0 (Pickering et al. 2011)	U.W. 88‐16; U.W. 88‐98
*Paranthropus robustus*	C	2.2–1.0 (Grine 1989; Gibbon et al. 2014; Kuman et al. 2021)	KW 6312; TM 1517, SKX 5017, SK 1813
*Paranthropus boisei*	B	2.5–1.3 (Walker et al. 1986; Kullmer et al. 1999; Dominguez‐Rodrigo et al. 2013)	KNM‐ER 1476; OH 8; KNM‐ER 1464
*Homo erectus* s.l. (Africa)	B, C	2.0–0.9 (Potts et al. 2004; Gathogo and Brown 2006)	Dmanisi
*Pan*	A	7.9—present^a^ (Moorjani et al. 2016)	—
*Gorilla*	A	10.8—present^a^ (Moorjani et al. 2016)	—
*Pongo*	D	15.9—present^a^ (Besenbacher et al. 2019)	—
*Homo sapiens*	A, B, C, D	0.3—present^a^ (Hublin et al. 2017)	

Abbreviations: A, Northcentral Africa; B, Eastern Africa; C, Southern Africa; D, Asia.

^a^ In the case of the living apes, the FAD refers to the inferred divergence date of the branch leading to the extant species.

### 2.2 Phylogenetic Trees

We used three phylogenetic trees inferred in a previous study (Sekhavati et al. 2025). The first tree was inferred without imposing any topological constraints on hominin relationships. In addition, two phylogenetic analyses constrained the position of *A. africanus* and *A. sediba*. In one analysis, *A. sediba* was placed as the sister taxon to *A. africanus* (Irish et al. 2013; Kimbel and Rak 2017). In the other, *A. sediba* was constrained as the sister to *Homo*, with *A. africanus* positioned outside the *Homo* + *Paranthropus* clade (Berger et al. 2010; Mongle et al. 2019). These three phylogenies are presented in Figures 1, 2, 3.

The trees were constructed using the fossilized birth‐death (FBD) model (Stadler 2010, Heath et al. 2014), implemented with RevBayes 1.2.3 (Höhna et al. 2016) and based on previously published craniodental data (Mongle et al. 2023). We modified the Lewis Mk model to create an ordered character model for characters that were marked as ordered in the original dataset. A discrete‐gamma model for the site rate model was used with four rate categories.

To compare character evolution with paleoenvironment, we determined the age of each node in the phylogenetic tree. A “relaxed clock” model for time calibration was used, specifically the uncorrelated lognormal (UCLN) model, which assumes that substitution rates for each branch are drawn from a lognormal distribution (Drummond et al. 2006). We ran a Markov chain Monte Carlo (MCMC) algorithm for 2 million generations across two chains, with parameters sampled every 2000 generations. Convergence and performance of the MCMC were assessed using Tracer 1.7.2 (Rambaut et al. 2018) to determine the effective sample size (ESS). The results were summarized by generating the maximum clade credibility tree (MCC).

### 2.3 Biogeographical Analyses

We divided Africa into three broad biogeographic regions following Strait's (Strait 2013) methodology: central Africa, eastern Africa, and southern Africa (Table 1). These regions correspond to our knowledge of the hominin fossil record and paleoenvironmental data. Additionally, since orangutans currently live on the islands of Borneo and Sumatra, we included another category, D, to represent Asia.

Biogeographic models, including DIVA (Ronquist 1997), DEC (Ree and Smith 2008), and BayArea (Landis et al. 2013), were compared using the R package BioGeoBEARS v. 1.1.2 (Matzke 2013). We assessed the relative fit of each model using the sample size‐corrected Akaike Information Criterion (AICc) (Burnham and Anderson 2002). Full details on the biogeographic processes and analyses are provided in Supporting Information S2.

### 2.4 Paleoenvironmental Proxies

After inferring the ancestral geographic range of each LCA, we used two main proxies to examine the possible association between paleoenvironmental change and major evolutionary events in postcranial morphology (i.e., the evolution of synapomorphies in LCAs; Table 2). For eastern Africa, we used stable carbon isotopes from paleosols to detect the regional records of major paleoenvironmental changes (17). Stable carbon isotopic ratios of paleosol carbonates distinguish between C4‐dominated and C3‐dominated ecosystems (Wynn 2000, 2004; Levin et al. 2011; Cotton et al. 2012) and have been widely used to study the paleoenvironment in eastern Africa (Wynn 2000, 2004; Levin et al. 2011; Cerling et al. 1988, 1991; Kingston et al. 1994; Sikes 1994; Pickford 1995; Retallack et al. 1995, 2002; Sikes et al. 1999; Quinn et al. 2007).

**TABLE 2:** List of inferred synapomorphies of various nodes in hominin phylogenies (49).

Node	Character	Character state	Function	Tree
LCA of *Australopithecus* and later hominins (Node 3)	Flexor hallucis longus groove orientation	Vertical	Everted ankle position relative to tibia (Latimer et al. 1987)	Tree 1 (0.78) Tree 2 (0.77) Tree 3 (0.84)
LCA of *Australopithecus* and later hominins (Node 3)	Talar axis angle	Low	Talocrural joint Eversion (DeSilva 2009)	Tree 1 (0.97) Tree 2 (0.71) Tree 3 (0.81)
LCA of *Australopithecus* and later hominins (Node 3)	Talar head torsion	High	Midtarsal stability (Manter 1941; Elftman 1960; Prang 2016a)	Tree 1 (0.71) Tree 2 (0.77) Tree 3 (0.71)
LCA of *Australopithecus africanus, Australopithecus sediba, Paranthropus*, and *Homo* (Node 4)	Lateral metatarsal diaphysis sagittal plane curvature	Straight	Lateral metatarsal stability (McNutt et al. 2018), a lack of forceful pedal flexion and presence of more human‐like push‐off	Tree 1 (0.63) Tree 2 (0.97)
LCA of *A. africanus, A. sediba, Paranthropus*, and *Homo* (Node 4)	Proximal phalanx curvature	Low curvature	Reduced phalangeal curvature associated with limited pedal grasping (Richmond 2007, Nguyen et al. 2014)	Tree 2 (0.73)
LCA of *A. africanus, A. sediba, Paranthropus*, and *Homo* (Node 4)	Proximal phalanx dorsal cant	Most canted	Increased metatarsophalangeal joint dorsiflexion (Latimer and Lovejoy 1990b)	**Tree 2 (0.55)** ^a^
LCA of *A. africanus, A. sediba*, and *Homo* (Node 5)	Proximal phalanx curvature	Flat	Associated with the absence of habitual grasping (Richmond 2007, Nguyen et al. 2014)	Tree 1 (0.90)
LCA of *A. africanus, A. sediba*, and *Homo* (Node 5)	Proximal phalanx dorsal cant	Most canting	Increased metatarsophalangeal joint dorsiflexion (Latimer and Lovejoy 1990b)	**Tree 1 (0.59)**
LCA of *Paranthropus*, *A. sediba*, and *Homo* (Node 6)	Cuboid facet angulation	Angled	Associated with longitudinal arch (Weidenreich 1923; Morton 1935)	**Tree 2 (0.50)**
LCA of *Paranthropus*, *A. sediba*, and *Homo* (Node 6)	Medial cuneiform Mt1 facet orientation	Distal	Hallux adduction (Prang 2016b)	Tree 2 (0.62)
LCA of *Paranthropus*, *A. sediba*, and *Homo* (Node 6)	Intermediate cuneiform proximodistal length	Intermediate	Intermediate midfoot elongation transforming the foot into a rigid lever	**Tree 2 (0.56)**
LCA of *Paranthropus*, *A. sediba*, and *Homo* (Node 6)	Mt1 robusticity	High	High medial foot loading (Aiello and Dean 1990)	Tree 2 (0.78)
LCA of *Paranthropus* and *Homo* (Node 8)	Medial cuneiform Mt1 facet orientation	Distal	Hallux adduction (Latimer and Lovejoy 1990a)	Tree 3 (0.65)
LCA of *Paranthropus* and *Homo* (Node 8)	Mt1 robusticity	High	High medial foot loading (Aiello and Dean 1990)	Tree 3 (0.67)
LCA of *A. africanus* and *A. sediba* (Node 9)	Calcaneus posterior talar facet shape	Convex	Subtalar joint mobility (Latimer and Lovejoy 1989; Prang 2016c)	**Tree 3 (0.59)**
LCA of *A. africanus* and *A. sediba* (Node 9)	Talar anterior calcaneal facet	Intermediate	Subtalar intermediate stability (Prang 2016c)	Tree 3 (0.60)
LCA of *Paranthropus* (Node 10)	Fibular facet flare	High	Heel strike foot posture of African apes (Gebo 1992)	Tree 1 (0.71) Tree 2 (0.78) Tree 3 (0.72)
LCA of *Paranthropus* (Node 10)	Trochlea keeling	High	Stabilize talocrural joint in species with diverse foot postures (Lewis 1980; Aiello and Dean 1990; DeSilva 2008)	Tree 1 (0.63) Tree 2 (0.74) Tree 3 (0.68)
LCA of *Homo* (Node 11)	Talar horizontal angle	Low	Transverse tarsal joint stability	Tree 1 (0.96) Tree 2 (0.97) Tree 3 (0.98)
LCA of *Homo* (Node 11)	Talar body height	Tall	Associated with longitudinal arch and/or decreased loading of ankle (Boyle and DeSilva 2015)	Tree 1 (0.98) Tree 2 (0.98) Tree 3 (0.95)
LCA of *Homo* (Node 11)	Talar posterior calcaneal facet angle	High	Increase subtalar joint stability (Prang 2016c)	**Tree 2 (0.59)** Tree 3 (0.62)
LCA of *Homo* (Node 11)	Orientation of mediolateral margins of the Mt1 head	Dorsoplantarly oriented	Associated with hallucal push‐off mechanism (Susman and Brain 1988)	Tree 1 (0.97) Tree 2 (0.96) Tree 3 (0.96)
LCA of *Homo* (Node 11)	Shape of plantar Mt1 head	Least angled	Metatarsophalangeal joint stability	Tree 1 (1.00) Tree 2 (0.94) Tree 3 (0.91)

*Note:* Boldface values indicate low probabilities relative to other traits and fewer trees.

Cerling et al. (2011) inferred paleoenvironmental conditions in eastern Africa. Their study estimated woody cover based on over 1300 published analyses of paleosols from hominin sites in the Awash and Omo‐Turkana basins over the past 7 million years. They used UNESCO classifications of African vegetation to determine the extent of woody cover. For our analysis, we grouped the estimates into three environmental categories (Table 3): wet, mixed, and dry. Wet environments were defined as having more than 40% woody cover, mixed environments had 30%–40% woody cover, and dry environments had less than 30% woody cover. These classifications are rough estimates and should be interpreted with caution.

**TABLE 3:** Eastern African paleoenvironmental intervals according to paleosol carbonates (Cerling et al. 2011).

Time (Mya)	7.4–5.5	5.5–4.2	4.2–2.94	2.94–1.87	1.87–0.83	0.83–0.64	0.64–0.16
Environment	Dry^a^	Mixed	Wet	Mixed	Dry	Mixed	Dry

^a^ Because time intervals are defined by changes in environmental conditions, a “dry” environment must have been preceded by a wetter environment (either mixed or wet).

Since high‐resolution paleosol data are unavailable for southern African hominin sites, we used Pickering et al.'s study (Pickering et al. 2019) on flowstone growth (Table 4). Massive flowstones, which form during periods of increased rainfall, correspond to wet periods (Pickering et al. 2019). Mixed periods correspond to cave breccias (flowstone bound sedimentary units) (Vrba 1985) traditionally interpreted as being deposited during mesic conditions (as indicated by faunal analysis). Dry periods correspond to cave breccias traditionally interpreted (Vrba 1985) as being deposited during xeric conditions (also indicated by faunal analysis). Pickering et al.'s (2019) study only covers up to 3.19 million years ago (mya) in South Africa. For time periods older than that, we used data from Diekmann et al. (2003) on the continental climate in southern Africa based on ocean floor sedimentology.

**TABLE 4:** Southern African paleoenvironmental intervals according to massive flowstones, flowstone bound sedimentary units in caves, and ocean floor sedimentology (Pickering et al. 2019; Diekmann et al. 2003).

Time (Mya)	~6.0–3.19^a^	3.19–3.08	3.08–2.83	2.83–2.62	2.62–2.28	2.28–2.17	2.17–2.12	2.12–2.0	2.0–1.82	1.82–1.63	1.63–1.41	1.41–1.32	< 1.32
Environment	Wet	Wet	Mixed	Wet	Mixed	Wet	Mixed	Wet	Dry	Wet	Dry	Wet	Dry

^a^ Data from Diekmann et al. (2003).

Although central Africa was not commonly inhabited by the taxa included in this study, we accounted for the possibility that some internal nodes might be associated with central Africa. In such cases, we used Novello et al. (2017) study, which dated vegetation reconstructions between 8 and 1 Ma in northern Chad. This study analyzed phytolith and diatom remains preserved at 45 Miocene and Pliocene localities to infer vegetation types. A summary of proxy type, time range, and source for each region is provided in Table 5.

**TABLE 5:** A summary of proxy type, time range, and source for each biogeographical region included in our study.

Region	Proxy type	Time coverage	Source
Eastern Africa	Stable carbon isotopes (paleosols)	Past 7 million years	Cerling et al. (2011)
Southern Africa	Flowstone growth and cave breccias	Up to 3.19 Ma (Pickering); older via ocean sediments	Pickering et al. (2019), Vrba (1985), Diekmann et al. (2003)
Central Africa	Phytoliths and diatom remains	8–1 Ma	Novello et al. (2017)

If an ancestral node was reconstructed to have inhabited more than one region (e.g., eastern and southern Africa) and the regions differed in environmental conditions (e.g., one dry, one wet), we classified the paleoenvironment as being both (e.g., wet and dry). We also examined whether each ancestral node was wetter or drier compared to its immediate predecessor. Two nodes may both occupy reconstructed “mixed” environments, yet one may follow a dry ancestor, indicating a shift toward wetter conditions, whereas the other follows a wet ancestor, indicating a shift toward aridity. These changes are ecologically meaningful and could reflect different types of pressure on locomotor traits.

## 3 Results

Here we examined whether the evolution of shared derived traits (synapomorphies) in hominin feet evolved consistently with specific environments. To do so, we collected paleoenvironmental data from various sources (Tables 3 and 4). We used tip‐dated phylogenies to estimate the divergence times of different hominin lineages. At each node, we reconstructed the regions in Africa occupied by hominin ancestors. Finally, we recorded the synapomorphies evolving at each LCA identified in a previous study (Sekhavati et al. 2025). Tables 6, 7, 8 summarize the results of tip dating, historical biogeography, paleoenvironmental conditions, and the number of synapomorphies. Figures 1, 2, 3 illustrate the results of historical biogeography and the reconstructed paleoenvironment at each node.

**TABLE 6:** A summary of the age estimates for each node, the ancestral range (with probabilities of lineage presence in specific regions shown in parentheses), the paleoenvironmental conditions (derived from Tables 3 and 4), and the number of synapomorphies (Table 2) for Tree 1.

Node	Node name	Node age (mya)	Number of synapomorphies	Ancestral range	Paleoenvironment	Direction of ecological change compared to prior ancestor
N1	LCA of *Homo + Pan*	8.3	0	Central (82%)	Wet	NA
N2	LCA of Hominins	6.8	0	Eastern (67%)	Dry	Drier than N1
N3	LCA of *Australopithecus* and later hominins	5.4	3	Eastern (40%)	Mixed	Wetter than N2
N4	LCA of *A. africanus, A. sediba, Paranthropus* and *Homo*	4.4	1	Southern (75%)	Wet	Wetter than N3
N5	LCA of *A. africanus, A. sediba* and *Homo*	3.7	2	Southern (96%)	Wet	No change compared to N4
N7	LCA of *A. sediba* and *Homo*	2.7	0	Southern (94%)	Wet	No change compared to N5
N10	LCA of *Paranthropus*	2.7	2	Southern (66%)	Wet	No change compared to N4
N11	LCA of *Homo*	1.8	5	Eastern‐Southern (97%)	Dry and Wet	Drier in E. Africa compared to N7

**TABLE 7:** A summary of the age estimates for each node, the ancestral range (with probabilities of lineage presence in specific regions shown in parentheses), the paleoenvironmental conditions (derived from Tables 3 and 4), and the number of synapomorphies (Table 2) for Tree 2.

Node	Node name	Node age (mya)	Number of synapomorphies	Ancestral range	Paleoenvironment	Direction of ecological change compared to prior ancestor
N1	LCA of *Homo + Pan*	8.1	0	Central (82%)	Wet	NA
N2	LCA of Hominins	6.5	0	Eastern (62%)	Dry	Drier than N1
N3	LCA of *Australopithecus* and later hominins	5.2	3	Central‐Eastern (35%)	Mixed	Wetter than N2
N4	LCA of *A. africanus, A. sediba, Paranthropus* and *Homo*	4.3	3	Southern (92%)	Wet	Wetter than N3
N6	LCA of *Paranthropus*, *A. sediba* and *Homo*	3.6	4	Southern (88%)	Wet	No change compared to N4
N7	LCA of *A. sediba* and *Homo*	2.8	0	Southern (92%)	Wet	No change compared to N6
N10	LCA of *Paranthropus*	2.4	2	Southern (76%)	Mixed	Drier than N6
N11	LCA of *Homo*	1.8	5	Eastern‐Southern (98%)	Dry and Wet	Drier in E. Africa compared to N7

**TABLE 8:** A summary of the age estimates for each node, the ancestral range (with probabilities of lineage presence in specific regions shown in parentheses), the paleoenvironmental conditions (derived from Tables 3 and 4), and the number of synapomorphies (Table 2) for Tree 1.

Node	Node name	Node age (mya)	Number of synapomorphies	Ancestral range	Paleoenvironment	Direction of ecological change
N1	LCA of *Homo + Pan*	8.4	0	Central (82%)	Wet	NA
N2	LCA of Hominins	6.8	0	Eastern (66%)	Dry	Drier than N1
N3	LCA of *Australopithecus* and later hominins	5.5	3	Eastern (39%)	Mixed	Wetter than N2
N4	LCA of *A. africanus, A. sediba, Paranthropus* and *Homo*	4.5	0	Southern (78%)	Wet	Wetter than N3
N8	LCA of *Paranthropus* and *Homo*	3.8	2	Southern (67%)	Wet	No change compared to N4
N9	LCA of *A. africanus* and *A. sediba*	3.6	2	Southern (100%)	Wet	No change compared to N4
N10	LCA of *Paranthropus*	2.5	2	Southern (63%)	Mixed	Drier than N8
N11	LCA of *Homo*	2.2	5	Eastern‐Southern (98%)	Mixed in Eastern Africa, Wet in Southern Africa	Drier in E. Africa compared to N8

### 3.1 Biogeographic Reconstruction

The relationship between environment and trait evolution could be inferred by studying where traits evolved. The best approximating model for each phylogenetic tree was BAYAREALIKE with founder event (BAYAREALIKE + j), largely consistent with a previous study of hominin biogeography (Sekhavati and Strait 2024). Most reconstructions were consistent across different trees. For each node, the most likely ancestral areas occupied by any given LCA are shown in Figures 1, 2, 3.

We have summarized in Tables 6, 7, 8 and Figures 1, 2, 3. Based on the best approximating model (BAYAREALIKE + j), Central Africa was identified as the most likely ancestral region for the LCA of *Homo* and *Pan* in all trees (Node 1). Additionally, eastern Africa was consistently identified as the most likely ancestral region for the LCA of Hominins (Node 2).

The reconstruction differs among the trees for the LCA of *Australopithecus* and later hominins (Node 3). According to Trees 1 and 3 (Figures 1 and 3), this ancestor occupied eastern Africa, while Tree 2 suggests it occupied both eastern and southern Africa. From Nodes 4 to 10, all ancestors are identified as southern African across all trees. Finally, for the LCA of *Homo* (Node 11), results also vary among trees. Tree 1 indicates a southern African origin, whereas Trees 2 and 3 suggest an origin in both southern and eastern Africa.

### 3.2 Reconstructed Paleoenvironment at Internal Nodes

Across all phylogenetic trees, the LCA of *Homo* and *Pan* (Node 1; Figures 1, 2, 3) is reconstructed in Central Africa under wet environmental conditions. No synapomorphies are inferred at this node (Tables 5, 6, 7).

Node 2 (the LCA of hominins) is inferred in Eastern Africa under dry environmental conditions (Figures 1, 2, 3 and Tables 5, 6, 7). These dry conditions represent a shift from the wet condition reconstructed at Node 1. No foot synapomorphies are inferred at this node.

Node 3 (the LCA of *Australopithecus* and later hominins) is reconstructed under mixed environmental conditions (Figures 1, 2, 3 and Tables 5, 6, 7). Relative to Node 2, this node reflects a shift toward wetter environments. At Node 3, the analysis infers three synapomorphies. Trees 1 and 3 place this ancestor in Eastern Africa, whereas Tree 2 supports a broader Central–Eastern African distribution. No pedal synapomorphies are inferred at this node.

Node 4 represents the LCA of *A. africanus, A. sediba, Paranthropus*, and *Homo* and is reconstructed in Southern Africa under wet conditions (Figures 1, 2, 3). Although different trees inferred between zero and three synapomorphies (Tables 5, 6, 7), they converge on the same ecological signal. Node 4 exhibits a clear trend toward wetter conditions relative to Nodes 3 and 2.

Node 5 (the LCA of *A. africanus* , *A. sediba*, and *Homo*) appears only in Tree 1 (Figure 1 and Table 5), whereas Node 6 (the LCA of *Paranthropus*, *A. sediba*, and *Homo*) appears only in Tree 2 (Figure 2 and Table 6). Node 5 exhibits two synapomorphies, and Node 6 exhibits four. Both ancestors were reconstructed in wet environments in Southern Africa. We did not find evidence for an environmental shift at these noted relative to their immediate predecessor. Their evolution coincides with periods of stable wet environments.

Node 7, the LCA of *A. sediba* and *Homo*, appears in Trees 1 and 2 and reconstructs this ancestor in a wet Southern African environment (Figures 1 and 2, and Tables 5 and 6). This reconstruction is similar to earlier nodes and indicates persistent wet conditions. No synapomorphies arise at this node, and we did not identify any ecological shift that distinguishes it from its immediate predecessors. This is similar to Tree 3 (Figure 3 and Table 7), where Nodes 8 and 9 appear. Node 8 represents the LCA of *Paranthropus* and *Homo*, and Node 9 represents the LCA of *A. africanus* and *A. sediba*. Both nodes place these ancestors in Southern Africa under wet conditions and associate each with two synapomorphies. However, at these nodes we did not find evidence for environmental transition. Instead, all southern nodes consistently show diversification within stable wet environments up until this point.

Node 10 is the LCA of *Paranthropus* (Figures 1, 2, 3 and Tables 5, 6, 7) and is reconstructed in Southern Africa across all trees. All trees infer two synapomorphies at this node. The environmental signal differs among phylogenetic trees. Based on Tree 1, this ancestral node lived in wet conditions, whereas Trees 2 and 3 reconstruct it as living during mixed environments. The mixed environment indicates a relative shift toward drier conditions compared with earlier wet nodes.

Lastly, the LCA of *Homo* (Node 11) is reconstructed in both Eastern and Southern Africa across all trees (Figures 1, 2, 3 and Tables 5, 6, 7). All trees infer five synapomorphies at this node, indicating a significant number of trait evolution. While morphological inference is consistent across topologies, environmental reconstructions vary. Trees 1 and 2 reconstruct a combination of dry and wet conditions, whereas Tree 3 reconstructs mixed conditions in Eastern Africa and wet conditions in Southern Africa. Node 11 therefore marks both geographic and ecological expansion within the evolutionary history of *Homo*.

### 3.3 Summary of Ecological Transitions and Trait Evolution

Our results suggest that most internal nodes were predominantly associated with wet environments (Nodes 1, 4–9, as well as Node 10 in Tree 1; Figures 1, 2, 3). Mixed environments were the second most common, occurring at Nodes 3 and 10 (only in Trees 2 and 3). Dry environments were the least common, observed only at Node 2. The combination of wet and dry conditions was found only at Node 11.

For each tree we observed the following patterns in the environmental conditions under which the various synapomorphies evolved. In Tree 1 (Figure 1), five synapomorphies evolved in wet environments (Nodes 4, 5, and 10; Table 5). Node 11 had five synapomorphies that evolved under a combination of wet and dry conditions. Three synapomorphies evolved in mixed environments at Node 3. In Tree 2 (Figure 2), seven synapomorphies evolved in wet environments at Nodes 4 and 6 (Table 6). Mixed environments at Nodes 3 and 10 are associated with five synapomorphies. Finally, another five synapomorphies evolved under dry and wet conditions at Node 11. In Tree 3 (Figure 3), 10 synapomorphies evolved in mixed environments at Nodes 3, 10, and 11. Four synapomorphies evolved in wet environments at Nodes 8 and 9. On average, across all trees, 5.3 synapomorphies evolved in wet environments, 5.0 in dry and wet environments, and 4.3 in mixed environments.

When examining the direction of ecological change compared to prior ancestors, the results showed that in Tree 1, Nodes 3 and 4 (with four synapomorphies) were wetter than their previous nodes. In contrast, Node 11 with five synapomorphies was drier only in eastern Africa. In Tree 2, Nodes 3 and 4 (with six synapomorphies) were wetter than their previous nodes. Node 10, with two synapomorphies, was drier than its predecessor. As before, Node 11, with five synapomorphies, was drier only in eastern Africa. Lastly, in Tree 3, Node 3, with three synapomorphies, was wetter than its previous node. In contrast, Node 10, with two synapomorphies, was drier than its preceding node. Node 11, with five synapomorphies, was again drier only in eastern Africa compared to its previous node.

When we combined the reconstructed environment together with the direction of ecological changes from the parents' nodes, we observed that in Tree 1, eight synapomorphies evolved in conditions that were wet or becoming wetter (e.g., a node with mixed environment that comes after a node with dry environment). In Tree 2, 10 synapomorphies evolved in conditions that were wet or becoming wetter. Two synapomorphies evolved when conditions were becoming drier. In Tree 3, seven synapomorphies evolved in conditions that were wet or becoming wetter, particularly in mixed environments following dry periods. Two synapomorphies evolved at Node 10 when it was becoming drier. In all trees, regarding Node 11, it is unclear whether these synapomorphies primarily evolved in eastern Africa as it became drier or in southern Africa in which the environment was wet.

## 4 Discussion

This study examines the timing, biogeography, and environmental conditions under which synapomorphies related to bipedalism in the hominin foot evolved. Our results suggest that the origin of bipedalism coincided with increasing aridity in Central and Eastern Africa. However, wetter environments were important later in hominin evolution, as the majority of derived traits evolved during periods characterized by wet or mixed conditions. We argue that bipedalism likely originated in relatively dry, open habitats at the base of the hominin lineage, followed by a shift toward wetter, more closed environments. Bipedalism evolved during a dry period, but derived pedal traits evolved at different times. This suggests that although the advent of bipedalism may have been associated with adaptations in the postcranial skeleton, the foot was not among the regions exhibiting such adaptations (Sekhavati et al. 2025).

The case of *A. ramidus* at Aramis (e.g., ARA‐VP‐6/500) remains central to debate between those that promote either the savannah or forest hypothesis. According to White et al. (2009, 2010) early hominins, including *Ardipithecus*, lived in densely wooded environments based on diverse faunal and isotopic evidence which better fits the forest hypothesis. In contrast, based on stable isotope data of paleosols sampled from a wide range of eastern African sites, Cerling et al. (2010, 2014) argue that *Ardipithecus* lived in more open environments with a low fraction of woody tree cover, such as tree‐ or bush‐savannahs, more in line with the Savannah Hypothesis. Levin et al. (2008) further contributes to this discussion with isotopic data from Gona, pointing to a bushland to grassland environment for *Ardipithecus*. Simpson et al. (2019) inferred a more derived foot function and terrestrial bipedalism in the Gona sample, such as a reduced talar angle and a slightly more dorsally domed first metatarsal head, compared to the Aramis sample of *A. ramidus*.

We reconstructed a dry environment at the base of the hominin clade wherein bipedalism originated. This supports Cerling et al. (2011) findings and is consistent with the Savannah Hypothesis, suggesting that open and arid landscapes might have provided selection pressures for the evolution of bipedalism. However, occupying such environments by early hominins does not imply abandoning arboreal behavior. Early hominins could still have used trees for foraging for preferred foods, constructing tree nests, avoiding potential predators, and so on.

The earliest hominins had numerous traits associated with vertical climbing and forelimb‐dominated suspension (Daver et al. 2022; Richmond and Jungers 2008; Lovejoy, Simpson, et al. 2009; Kozma et al. 2018; Prang et al. 2021; Meyer et al. 2023; Araiza 2025). For example, the radius of *A. ramidus* and the ulna of *Sahelanthropus tchadensis* each has African ape‐like morphology that reflects climbing and/or knuckle‐walking ancestry (Prang et al. 2021; Meyer et al. 2023). The forelimbs of *Ardipithecus* and *Orrorin* include significantly curved manual phalanges and large distal humeri with prominent brachioradialis attachments that are associated with using the forelimb for climbing and/or suspension (Richmond and Jungers 2008; Lovejoy, Simpson, et al. 2009; Prang et al. 2021; Haile‐Selassie 2001). The ARA‐VP‐6/500 partial skeleton of *A. ramidus* includes a medial cuneiform with a medially oriented first metatarsal facet, which indicates the presence of a divergent hallux. Thus, although the origin of bipedalism might be associated with drier, more open environments, this reconstruction does not imply that hominins abandoned arboreal locomotion.

The reconstructed environments of the LCAs of hominins like *Australopithecus* and *Paranthropus* shifted toward wetter, more forested habitats. This result suggests that bipedalism was not restricted to arid environments. Paleoanthropologists have debated the functional and adaptive significance of ape‐like traits in *Australopithecus* over several decades (Ward 2002; Stern 2000). It is possible that bipedal traits were initially selected in drier, more open environments in the hominin LCA (Dart 1925, Cerling et al. 2011), then a shift toward wetter and more closed environments led to increased arboreality among taxa such as *A. afarensis*, *A. africanus* , and *A. sediba* (Prang 2016c, 2015; McHenry and Berger 1998; Green et al. 2007; Zipfel et al. 2011; Prabhat et al. 2021). The presence of climbing‐related traits in geologically younger taxa challenges a simple, unidirectional model of increasingly terrestrial bipedalism. Furthermore, recent phylogenetic analyses suggest the presence of homoplasy in pedal characters that reflect climbing (Sekhavati et al. 2025).

Recent footprints provide evidence for hominin locomotor diversity in the Turkana Basin, reflecting differences between *Paranthropus* and *Homo* (Hatala et al. 2024). Foot and other postcranial fossils from the Omo‐Turkana Basin are consistent with morphological and functional diversity. This might suggest increased arboreality among specimens attributed to *P. boisei* (Wood 1974, Gebo and Schwartz 2006, Boyle and DeSilva  2015; Ward et al. 2023).

Importantly, the Omo talus (OMO 323‐76‐898) and the Koobi Fora tali (KNM‐ER 1464 and KNM‐ER 1476) are not necessarily contemporaneous and may be separated by several hundred thousand years (Feibel et al. 1989; Gathogo and Brown 2006). We do not interpret these specimens as representing a direct evolutionary progression. Rather, we compare their morphologies to highlight variation within the Omo–Turkana Basin. The KNM‐ER 1464 and KNM‐ER 1476 tali, hypothesized to represent *P. boisei*, have increased trochlear grooving and a flaring lateral malleolar facet, similar to gorillas, as opposed to the more *Homo*‐like OMO 323‐76‐898 talus (Gebo and Schwartz 2006). Increased trochlear grooving and lateral malleolar flaring are associated with enhanced ankle mobility (Lewis 1980; Aiello and Dean 1990; Gebo 1992; DeSilva 2008) relative to their reconstructed ancestral condition. Greater ankle capacity is typically associated with arboreal behaviors.

The morphology of the OH 8 talus, initially attributed to *Homo habilis* (Leakey et al. 1964) but more recently suggested to represent *P. boisei* (Wood and Constantino 2007; DeSilva et al. 2019), is comparable to that of KNM‐ER 1464 and KNM‐ER 1476. The first metatarsals from Swartkrans hypothesized to represent *P. robustus* (SKX 5017, SK 1813) have a primitive morphology more similar to *A. afarensis* specimens from Hadar (A.L. 333‐21, A.L. 333‐115) than Sterkfontein *Australopithecus* (StW 562, StW 595) or Dmanisi *Homo* (Susman and Brain 1988; Susman and De Ruiter 2004; Pontzer et al. 2010). Although primitive‐like, these character states are reconstructed as synapomorphies of the *Paranthropus* clade and are therefore interpreted as representing derived morphologies rather than retained pleisomophies.

We caution that ape‐like morphology does not necessarily imply habitual arboreality. As emphasized recently (Mongle et al. 2025), *Paranthropus* postcrania show convergence with great apes. These features may be in response to multiple functional demands and may not uniquely diagnose climbing behavior. Thus, while increased ankle mobility is associated with arboreal positional behaviors in primates, the locomotor behavioral of *Paranthropus* remains uncertain. We argue that although these pedal characters, combined with other postcranial traits (e.g., the large upper limb of OH 80) (Dominguez‐Rodrigo et al. 2013), are consistent with increased arboreal capability among *Paranthropus*, they do not provide proof of habitual arboreality.

The functional and taxonomic implications of early *Homo* postcrania have been debated for decades (Wood and Collard 1999; Bramble and Lieberman 2004). The LCA of *Homo* likely lived in both dry and wet environments. Arid periods have been documented in key regions, such as the Turkana Basin in eastern Africa. These repeated aridification events (Wynn 2004; Boës et al. 2019; Yost et al. 2021) suggest that *Homo* species were well‐adapted to changing environments.

Regarding morphological diversity, there are two postcranial morphology among early east African *Homo*. One is best represented by OH 62 (Johanson et al. 1987) and KNM‐ER 3735 (Leakey and Walker 1985), attributed to *H. habilis*. The other is represented by KNM‐WT 15000 (Brown et al. 1985), attributed to *H. erectus* . The presence of these two morphologies likely reflects locomotor diversity among these taxa (Ward et al. 2015). OH 62 and KNM‐ER 3735 have relatively large upper limbs, possibly indicating the retention of forelimb dominated arboreality among *H. habilis* (Ruff 2009, Haeusler and McHenry 2007). KNM‐WT 15000, despite its juvenile status, has much more human‐like limb proportions (e.g., Prabhat et al. 2021). The foot elements from Dmanisi are incomplete, but they indicate an adducted hallux, everted ankle, and a well‐developed transverse arch (Pontzer et al. 2010), suggesting more efficient bipedal walking and running in *Homo*, as reflected by KNM‐WT 15000. The combination of morphological diversity in early *Homo* is intriguing, as dry and wet environments were both occupied by them.

The synapomorphies we previously identified (Sekhavati et al. 2025) evolved in closed or mixed environments (i.e., the mixed environment that were more closed than their ancestral habitats). Only a small number of traits evolved in dry conditions. Although most synapomorphies evolved in closed (wet) conditions, descendants may have retained these traits even in environments different from their ancestors'. This suggests ecological flexibility in early hominins, where traits advantageous in one environment remain beneficial or neutral in another. Finally, our results show that hominin foot evolution was not linear (DeSilva et al. 2019; Sekhavati et al. 2025).

### 4.1 Regional and Site‐Level Paleoenvironmental Signals

Regional scale paleoenvironmental data provides strong framework for understanding the environmental context of hominin evolution. These broad ecological patterns derived from time‐averaged data across long intervals limit our ability to identify habitat preferences within mosaic landscapes. This limitation prevents high‐resolution ecological data reconstruction at small geographical scales. But we need broad regional trends to reconstruct the environmental context at a regional scale.

Paleoenvironmental data from specific sites are still important for our inference. This is especially true for *S. tchadensis*. Although this species is central to the discussions of the origin of bipedalism (Williams et al. 2026), we could not include it in our study because it lacks foot bones. We may never be able to identify the exact site where bipedalism evolved, but the paleoenvironmental evidence from the *S. tchadensis* offers important insight. The type locality of *S. tchadensis* (TM 266) had considerable tree cover and comprised a mosaic of wooded areas (Vignaud 2002; Novello et al. 2017). This reconstruction of a wooded landscape supports our conclusion that the LCA of *Pan* and *Homo* lived in relatively wet heterogeneous environments.

Analyzing site‐level paleoenvironment is important, but large‐scale environmental data reveal ecological pressures that shape evolution. These data capture ecological changes across thousands of years. For example, Central Africa recorded a sustained shift from wooded environments to more open grasslands between ~8 and 3 Ma (Novello et al. 2017). This suggests progressive drying at the regional scale. Our data suggest that bipedalism evolved during a shift from wetter to drier conditions. This also aligns with site‐level paleoenvironmental evidence from northern Chad, where hominins appear during intervals of increasing aridity (Novello et al. 2017).

A similar pattern appeared in East Africa, where we reconstructed several nodes, including the LCA of *Ardipithecus* and later hominins. At Lukeino in Kenya (~6 Ma), associated faunal remains indicate that *Orrorin tugenensis* inhabited an open woodland with substantial tree cover (Pickford and Senut 2001; Senut et al. 2001). The regional environment gradually shifted toward drier conditions. Bipedalism evolved during this ecological transition. By the time of Aramis, habitats appear more open, with increased grassy vegetation (Cerling et al. 2010). The transition from wooded mosaics to more open environments signal the emergence of bipedalism in progressively drier conditions.

### 4.2 Taxonomic Uncertainty

Several foot bones are not directly associated with craniodental remains, which complicates their taxonomic attribution (Grine et al. 2022). We therefore based their attribution on published comparative anatomy. For example, we assigned OH 8, KNM‐ER 1464, and KNM‐ER 1476 to *P. boisei* based on pedal features that differ from contemporaneous *Homo* fossils (Gebo and Schwartz 2006; DeSilva et al. 2019; Wood and Constantino 2007).

Two functionally distinct talar morphotypes were found in the Turkana Basin. Thee primitive‐like morphotype (KNM‐ER 1464, KNM‐ER 1476, and KNM‐ER 5428) has generally been attributed to *Paranthropus*, and the more derived morphotype (Omo 323‐76‐898, KNM‐ER 813) to *Homo*. KNM‐ER 1464 and KNM‐ER 1476 likely belong to the same species because they share key talar features. These features include an asymmetrically grooved trochlea, flaring malleolar facets, more parallel anterior and posterior calcaneal facets, and a more ape‐like subtalar morphology (Gebo and Schwartz 2006; Boyle and DeSilva  2015; Prang 2016b). KNM‐ER 1464 also displays a deep, obliquely oriented flexor hallucis longus groove. This region is missing from KNM‐ER 1476, but the preserved portions of the two tali are closely similar.

OH 8 shows greater similarity to *Paranthropus* than to *Homo*. Its talus has a grooved and asymmetric trochlea, a feature found in specimens assigned to *Paranthropus*. In overall morphology, OH 8 is very similar to TM 1517, which was assigned to *P. robustus* . It also shares important features with KNM‐ER 1464 and KNM‐ER 1476. On the other hand, OH 8 does not resemble the more derived, *Homo*‐like tali such as Omo 323‐76‐898 and KNM‐ER 813 (Gebo and Schwartz 2006; Wood and Constantino 2007; DeSilva et al. 2019).

### 4.3 Limitations

Several factors limit our knowledge of hominin evolution and ecology. Most hominin taxa are known from only a few specimens (Su and Harrison 2008). Biases in fossil preservation (Behrensmeyer and Reed 2013) and uneven sampling (Maxwell et al. 2018) influence our understanding of their temporal and geographical distribution. Fossils are assigned to certain environments based on where the remains were found, not the habitats they lived in. Moreover, hominins usually make up a small part of fossil assemblages because of preservation and sampling biases (Maxwell et al. 2018; Behrensmeyer and Reed 2013; Su and Harrison 2008). In addition, the geographic distribution of fossil data is uneven, in part due to historical inequities in paleontological research, including the legacy of colonialism (Faith et al. 2021; Raja et al. 2022; Wood and Smith 2022; Monarrez et al. 2022). Finally, taxonomic uncertainty (Wood and Boyle 2016; Grine et al. 2022) and unresolved phylogenetic relationships (Mongle et al. 2019, 2023) introduce further challenges when assigning traits to specific lineages or nodes.

There are additional challenges associated with incorporating paleoenvironmental data (Baum and Larson 1991; Koehl 1996; Larson and Losos 1996; Thorpe et al. 2014). For our analysis, we used broad categories such as “wet” or “dry.” This simplification cannot capture the complexity and ecological variation. Large scale paleoenvironmental data still offers meaningful insights. Rather than reconstructing fine‐scale habitats, we identified broad‐scale associations between foot synapomorphies and environmental conditions. While site‐specific reconstructions are valuable, they often lack the information needed to address macroevolutionary questions. The questions we are interested in this paper differ from those typically addressed in high‐resolution paleoecological studies.

Therefore, we used environmental proxies that capture broad regional signals to answer evolutionary questions. Stable carbon isotope data from Cerling et al. (2011), for example, span multiple eastern African sites and provide an overview of vegetation over time. Flowstone records from Pickering et al. (2019) capture climate‐driven hydrological variation across several southern African cave sites. Although these records are discontinuous, they are geographically distributed and reflect regional wet–dry cycles. In central Africa, phytolith and diatom records from Novello et al. (2017) similarly track broad vegetation patterns.

When different types of data are available across regions, combining different proxies is challenging. Excluding data and choosing one proxy type would limit our phylogenetic and geographical analysis. Our proxies are appropriate for testing correlations between evolutionary changes and regional environmental trends due to their scale and nature.

## 5 Conclusion

Our results suggest that the *Homo*‐*Pan* LCA evolved during a wet period, while the LCA of hominins evolved during a dry period. This is supported by the shift from a wet environment to a drier, more open environment during the terminal Miocene (Zollikofer et al. 2005; Richmond and Jungers 2008; Lovejoy, Simpson, et al. 2009; Cerling et al. 2011; Daver et al. 2022). Most synapomorphies in hominin feet evolved in closed (wet) environments or those that became more closed during the Pliocene. The LCAs of most subsequent hominins were reconstructed in wet and mixed environments. These reconstructions are compatible with the presence of climbing‐related traits in some taxa. The LCA of *Homo* occupied both dry and closed environments prior to a shift toward more efficient bipedal walking and running. The persistence of pedal synapomorphies over time and across different ecological settings suggests hominins' flexibility to adapt to different environments. Additional datasets on different anatomical regions and taxa could provide deeper insight into these patterns in future studies. This study underscores the importance of phylogenetic information and paleoenvironmental data for understanding the evolution of bipedalism.

## Author Contributions

**Yeganeh Sekhavati:** conceptualization, investigation, funding acquisition, writing – original draft, methodology, validation, visualization, writing – review and editing, software, formal analysis, project administration. **Thomas Cody Prang:** conceptualization, investigation, writing – review and editing, supervision, writing – original draft. **David Strait:** conceptualization, investigation, funding acquisition, writing – review and editing, supervision, resources.

## Funding

This work was supported by the Wenner‐Gren Foundation (Grant No. 10244) and Biological Anthropology Directorate of the National Science Foundation (NSF) Biological Anthropology Program, within the Directorate for Social, Behavioral and Economic Sciences (BCS‐2141883).

## Supporting information


**Data S1:** Supporting Information.

## Data Availability

The data that support the findings of this study are openly available in github at https://github.com/yeganehsekhavati.
