# Supplementary material for: Environmental Context of Hominin Foot Evolution
Source: Am J Biol Anthropol. 2026 Oct 2;191(2):e70340. doi: 10.1002/ajpa.70340 (PMC13633444; doi:10.1002/ajpa.70340)
Supplement: Supplementary file 1 [file AJPA-191-e70340-s001.docx]

**Supplementary Information**

**S1.**

Note that Martin et al. (Martin et al., 2021) have argued that the specimens conventionally attributed to *A. sediba* cannot as yet be shown to represent a lineage independent from *A. africanus*, and thus the former should be allocated to *A. africanus sediba*. This issue is more than semantic, because the conventional taxonomy assumes that the Malapa hominins are fully independent entities in phylogenetic and biogeographic analyses, whereas the revised taxonomy does not. In the present analysis, the Malapa hominin were treated as an independent unit, and thus are recognized at the species level. Certain of the results of the present study are thus dependent on such a taxonomy.

**S2.**

**Biogeographic Processes**

The most fundamental processes in historical biogeography are dispersal, extinction, vicariance, and duplication (Matzke, 2013). Dispersal or range expansion is the process of expanding the geographic range caused by an organism's movement to a new area (e.g., when a species in area A is subsequently found in areas A and B [A→AB]). "Founder event" or "jump dispersal" is a particular case of range expansion in which one daughter lineage occupies the ancestral geographic range, whereas the other daughter lineage rapidly disperses to a new range outside the range of the ancestor (e.g., AB→AB, C). Local extinction or range contraction is the reverse process that results in the disappearance of a lineage from a region it once occupied (e.g., AB→A).

Vicariance, as may occur during allopatric speciation, is associated with splitting the range of the ancestral species. Variance can be narrow (in which one daughter species must occupy only a single region [e.g., ABC→AB, C]) or widespread (also called classical vicariance, in which both daughters occupy more than one area (e.g., ABCD→AB, CD). Finally, duplication, or sympatry, corresponds to within-area (intraregional) speciation.

Similar to vicariance, sympatry (Matzke, 2013) could be narrow (within-area duplication in which the ancestor and its descendants occupy a single area [e.g., A→A, A]) or widespread (within-area speciation in which the ancestor occupies more than one area [e.g., ABC→ABC, ABC]). Additionally, one daughter lineage could occupy a single area, whereas the other occupies the entire ancestral range (e.g., ABC→A, ABC). The last process is called subset sympatry, which corresponds to peripatric speciation. Although the terms mentioned above such as sympatry and allopatry have particular meanings in the speciation literature, they refer here to spatial biogeographical processes (e.g., sympatry refers in this case to geographic regions rather than narrow habitats).

**Biogeographical Analyses**

DIVA is a parsimony method. Regarding cladogenetic events, it allows narrow sympatry as well as narrow and widespread vicariance (Ronquist, 1997). DIVA uses a cost matrix to count biogeographical events, assigning a cost of one for extinctions and dispersals, while vicariance and duplication within a single area (narrow sympatry) have zero costs. The most parsimonious solutions are hypotheses that minimize dispersal events because they explain the data at the lowest cost.

Ree et al. (Ree et al., 2005) proposed a maximum likelihood model, later refined by Ree and Smith (Ree and Smith, 2008), known as dispersal-extinction-cladogenesis (DEC). DEC is a parametric model that assigns probabilities, rather than costs, to events. Anagenetic events (i.e., dispersal and extinction) are treated as free parameters that evolve along the branches of the phylogenetic tree. The instantaneous rate matrix is a matrix that represents the transition rate between discrete geographical ranges along phylogenetic branches.

Regarding cladogenetic events, both DIVA and DEC permit narrow sympatry and narrow vicariance but do not allow widespread sympatry (Ronquist, 1997, Ree and Smith, 2008). However, the two models differ in two significant ways. First, DIVA permits widespread vicariance, while DEC assumes that one of the daughter lineages must always have a range confined to a single area, thus prohibiting widespread vicariance (Ronquist, 1997, Ree and Smith, 2008). In addition, unlike DIVA, DEC allows subset sympatry (Ronquist and Sanmartín, 2011)

Finally, the BayArea model (Landis et al., 2013) assumes that ancestral ranges remain the same during cladogenesis. This model permits widespread and narrow sympatry. BayArea allows widespread sympatry, unlike DIVA and DEC.

These models include two free parameters: the rate of range expansion or dispersal (parameter d), and the rate of range contraction or extinction (parameter e) along the branches of the phylogenetic tree. For each tree, three additional models were created by incorporating 'founder-event' speciation events (parameter j), creating DIVALIKE+j, DEC+ j, BAYAREALIKE+ j models. Finally, we added an additional parameter to estimate the effect of distance on dispersal (i.e., whether the probability of dispersal increases as distance decreases [parameter x]). The distances between geographical areas were estimated as the minimum distance measured in kilometers using FreeMapTools (<http://www.freemaptools.com/how-far-is-it-between.htm>). Finally, we assessed the relative fit of each model using the sample size-corrected Akaike Information Criterion (AICc) of each model (Burnham and Anderson, 2002).

**References**

BURNHAM, K. P. & ANDERSON, D. R. 2002. *Model selection and multimodel inference,* New York, Springer.

LANDIS, M. J., MATZKE, N. J., MOORE, B. R. & HUELSENBECK, J. P. 2013. Bayesian analysis of biogeography when the number of areas is large. *Syst Biol,* 62**,** 789-804.

MARTIN, J. M., LEECE, A., NEUBAUER, S., BAKER, S. E., MONGLE, C. S., BOSCHIAN, G., SCHWARTZ, G. T., SMITH, A. L., LEDOGAR, J. A. & STRAIT, D. S. 2021. Drimolen cranium DNH 155 documents microevolution in an early hominin species. *Nature Ecology & Evolution,* 5**,** 38-45.

MATZKE, N. J. 2013. Probabilistic historical biogeography: new models for founder-event speciation, imperfect detection, and fossils allow improved accuracy and model-testing. *Frontiers of Biogeography,* 5.

REE, R. H., MOORE, B. R., WEBB, C. O. & DONOGHUE, M. J. 2005. A likelihood framework for inferring the evolution of geographic range on phylogenetic trees. *Evolution,* 59**,** 2299-2311.

REE, R. H. & SMITH, S. A. 2008. Maximum likelihood inference of geographic range evolution by dispersal, local extinction, and cladogenesis. *Syst Biol,* 57**,** 4-14.

RONQUIST, F. 1997. Dispersal-vicariance analysis: a new approach to the quantification of historical biogeography. *Systematic biology,* 46**,** 195-203.

RONQUIST, F. & SANMARTÍN, I. 2011. Phylogenetic Methods in Biogeography. *Annual Review of Ecology, Evolution, and Systematics,* 42**,** 441-464.
